# Late-Onset Intermittent Fasting Decreases Aging-Related Frailty

**DOI:** 10.1093/geroni/igaa057.411

**Published:** 2020-12-16

**Authors:** Yoko Henderson, Nazmin Bithi, Christopher Link, Jie Yang, Christopher Hine

**Affiliations:** 1 Cleveland Clinic Lerner Research Institute, Cleveland, Ohio, United States; 2 Lerner Research Institute, Cleveland Clinic, Cleveland, Ohio, United States; 3 Cleveland Clinic, Cleveland, Ohio, United States

## Abstract

Global average life expectancy continues to rise. As aging increases likelihoods of exhibiting geriatric syndromes (a.k.a. frailty), there is a need for effective anti-aging treatments. Multiple studies have shown the positive effects of dietary restriction (DR) on lifespan in various model organisms. However, DR is not widely implemented in older adults due to issues with patient compliance and the overall lack of understanding on the effects of DR initiated later in life. Thus, the present study tested whether late-life DR, specifically Every-Other-Day (EOD) fasting, attenuates aging-related frailty using a modified and simplified frailty index in mice. Briefly, 20-month old male and female C57BL/6 mice (human equivalent of 65 years) that had been on a control chow diet ad libitum during adulthood were placed on EOD fasting or ad libitum feeding for 2.5 months. Their frailty index was identified using an indirect calorimeter, glucose tolerance test, novel object place recognition test, forelimb grip strength meter, and rotarod. We found that late-life EOD fasting decreased overall caloric intake in males but not in females. In addition, EOD fasting significantly improved metabolic, musculoskeletal, and cognitive endpoints in male mice, but enhanced only some of these in female mice. Furthermore, EOD fasting improved hydrogen sulfide (H2S) production capacity and its associated sulfhydration signaling in tissues, which positively correlated with improvements in frailty measures. We conclude that EOD fasting implemented late in life can have therapeutic potential in the clinic. We are currently investigating the necessity of H2S production for DR mediated benefits and longevity.

